# A primordial ^15^N-depleted organic component detected within the carbonaceous chondrite Maribo

**DOI:** 10.1038/s41598-020-77190-z

**Published:** 2020-11-20

**Authors:** Christian Vollmer, Jan Leitner, Demie Kepaptsoglou, Quentin M. Ramasse, Ashley J. King, Paul F. Schofield, Addi Bischoff, Tohru Araki, Peter Hoppe

**Affiliations:** 1 Institut für Mineralogie, Westfälische Wilhelms-Universität, Corrensstr. 24, 48149 Münster, Germany; 2 Particle Chemistry Department, Max Planck Institute for Chemistry, Hahn-Meitner-Weg 1, 55128 Mainz, Germany; 3 SuperSTEM Laboratory, Keckwick Lane, Daresbury, WA4 4AD UK; 4 Jeol Nanocentre and Department of Physics, University of York, Heslington, YO10 5DD UK; 5 School of Chemical and Process Engineering and School of Physics and Astronomy, University of Leeds, Leeds, LS2 9JT UK; 6 Planetary Materials Group, Department of Earth Sciences, Natural History Museum, London, SW7 5BD UK; 7 Institut für Planetologie, Westfälische Wilhelms-Universität, Wilhelm-Klemm-Str. 10, 48149 Münster, Germany; 8 Diamond Light Source, Didcot, OX11 0DE UK

**Keywords:** Asteroids, comets and Kuiper belt, Early solar system, Meteoritics

## Abstract

We report on the detection of primordial organic matter within the carbonaceous chondrite Maribo that is distinct from the majority of organics found in extraterrestrial samples. We have applied high-spatial resolution techniques to obtain C-N isotopic compositions, chemical, and structural information of this material. The organic matter is depleted in ^15^N relative to the terrestrial value at around δ^15^N ~ -200‰, close to compositions in the local interstellar medium. Morphological investigations by electron microscopy revealed that the material consists of µm- to sub-µm-sized diffuse particles dispersed within the meteorite matrix. Electron energy loss and synchrotron X-ray absorption near-edge structure spectroscopies show that the carbon functional chemistry is dominated by aromatic and C=O bonding environments similar to primordial organics from other carbonaceous chondrites. The nitrogen functional chemistry is characterized by C-N double and triple bonding environments distinct from what is usually found in ^15^N-enriched organics from aqueously altered carbonaceous chondrites. Our investigations demonstrate that Maribo represents one of the least altered CM chondrite breccias found to date and contains primordial organic matter, probably originating in the interstellar medium.

## Introduction

Carbonaceous chondrites provide important samples of the very early solar nebula. These complex rocks recorded snapshots of events 4.57 Ga ago that can be disentangled by advanced analytical techniques on Earth. One of the most challenging components to analyze within such chondrites is the so-called “matrix”, a fine-grained mixture of presolar dust grains, amorphous and crystalline silicates, organic matter (OM), sulfides, and metal, in which larger constituents such as chondrules or refractory inclusions are embedded. OM and amorphous silicates within the carbonaceous chondrite matrix are especially important, because they are prone to alteration and destruction, and record crucial condensation and synthesis processes at low temperatures in the solar nebula and meteorite parent bodies^1–4^. Usually, they are analyzed within generally more primitive meteorites such as the “Renazzo-type” (CR) chondrites^5–7^, Wild 2 cometary material^8^, or chondritic-porous interplanetary dust particles (IDPs)^9,10^. However, it is well known that the aqueous and thermal alteration characteristics of CR chondrites and cometary dust are rather complex and highly heterogeneous on a sub-µm scale^4–10^. Still, these extraterrestrial samples provide the best reservoirs to investigate the most primordial dust and OM within the solar nebula. In contrast, “Mighei-type” (CM) chondrites are typically more aqueously altered than CR chondrites and IDPs and thus have recorded different alteration or evolutionary stages of OM. The CM chondrite Paris (of unknown origin; find, 2001) does contain some regions that show signs of strong alteration, whereas others seem to be less altered and resemble certain more altered CR chondrite matrix lithologies such as in Renazzo^11,12^. However, even the most unaltered regions within more primitive CM chondrites such as Paris are still more altered than the primitive lithologies of CR chondrites. Furthermore, most CM chondrites are breccias that consist of lithic clasts with different alteration stages. The observation that these lithic clasts still show their original accretionary texture indicates that a single starting material was affected by liquid water under different alteration conditions, followed by impact brecciation and mixing^13^. Therefore, evidence for truly primitive, i.e., minimally aqueously altered CM matrix and OM remains sparse^14,15^.

Here we have investigated fine-grained OM within less altered matrix regions of the CM chondrite Maribo, an observed fall from Denmark in 2009. This carbonaceous chondrite shows some unusual alteration characteristics different from other CM and CR chondrites^16,17^. Furthermore, recent transmission electron microscopy (TEM) and nano-scale secondary ion mass spectrometry (NanoSIMS) work supports the relatively unaltered character for a CM chondrite^18^. We show that the Maribo matrix contains a primordial OM component with a strongly ^15^N-depleted isotopic signature that is rare among chondritic OM and has not been described in chemical detail in other extraterrestrial samples to date. The functional chemistry signature of this unique material, obtained by electron energy loss spectroscopy (EELS) and synchrotron X-ray spectroscopy methods, demonstrates the relatively unaltered nature of this OM. This organic component apparently has recorded a snapshot of very early organic chemistry otherwise obscured in other primitive carbonaceous chondrites by more severe alteration processes. Our combined high-spatial resolution analyses of primitive matrix components therefore highlight the important character of Maribo as a key sample of the very early solar nebula.

## Results

**Figure 1:**
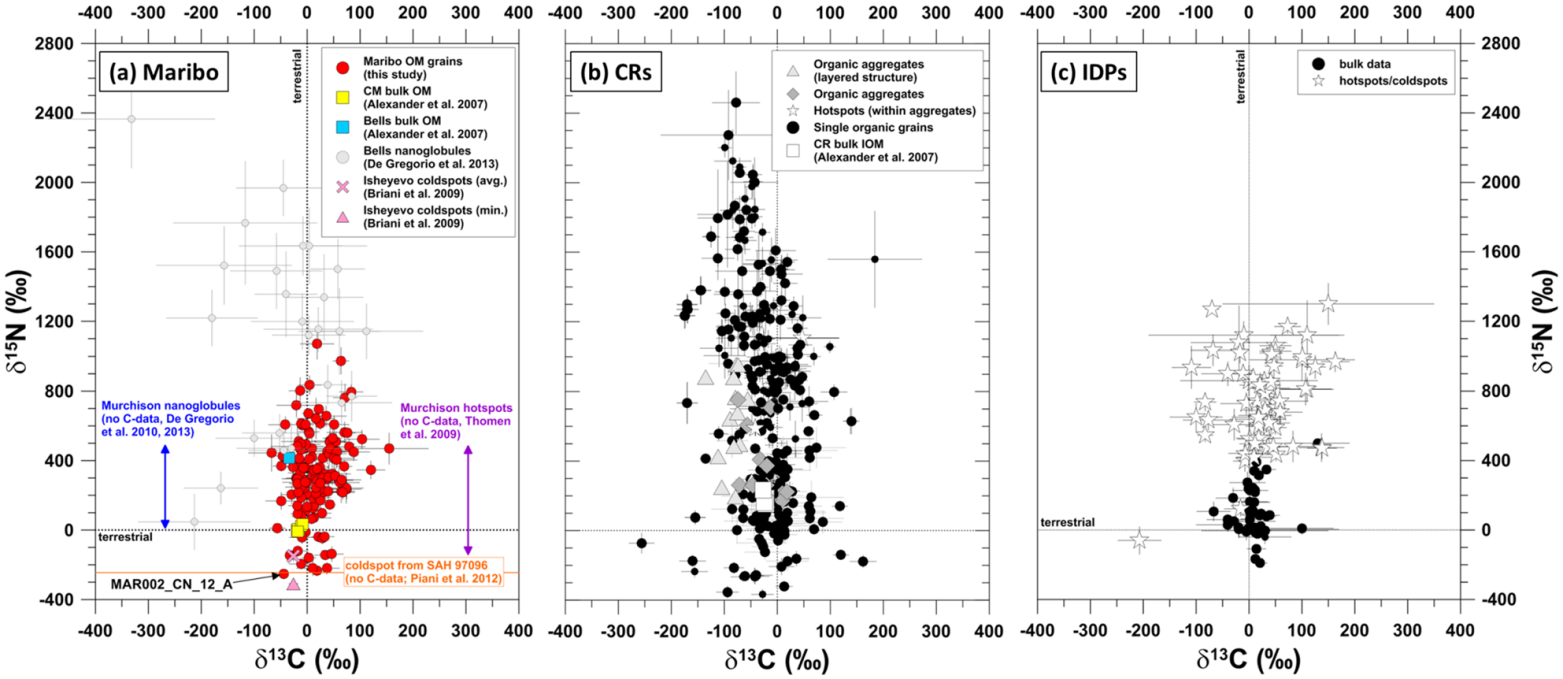
Nitrogen and carbon isotopic compositions of Maribo OM compared to CR, CM, and IDP organic materials from both bulk and in-situ investigations. See text for references.

**Figure 2:**
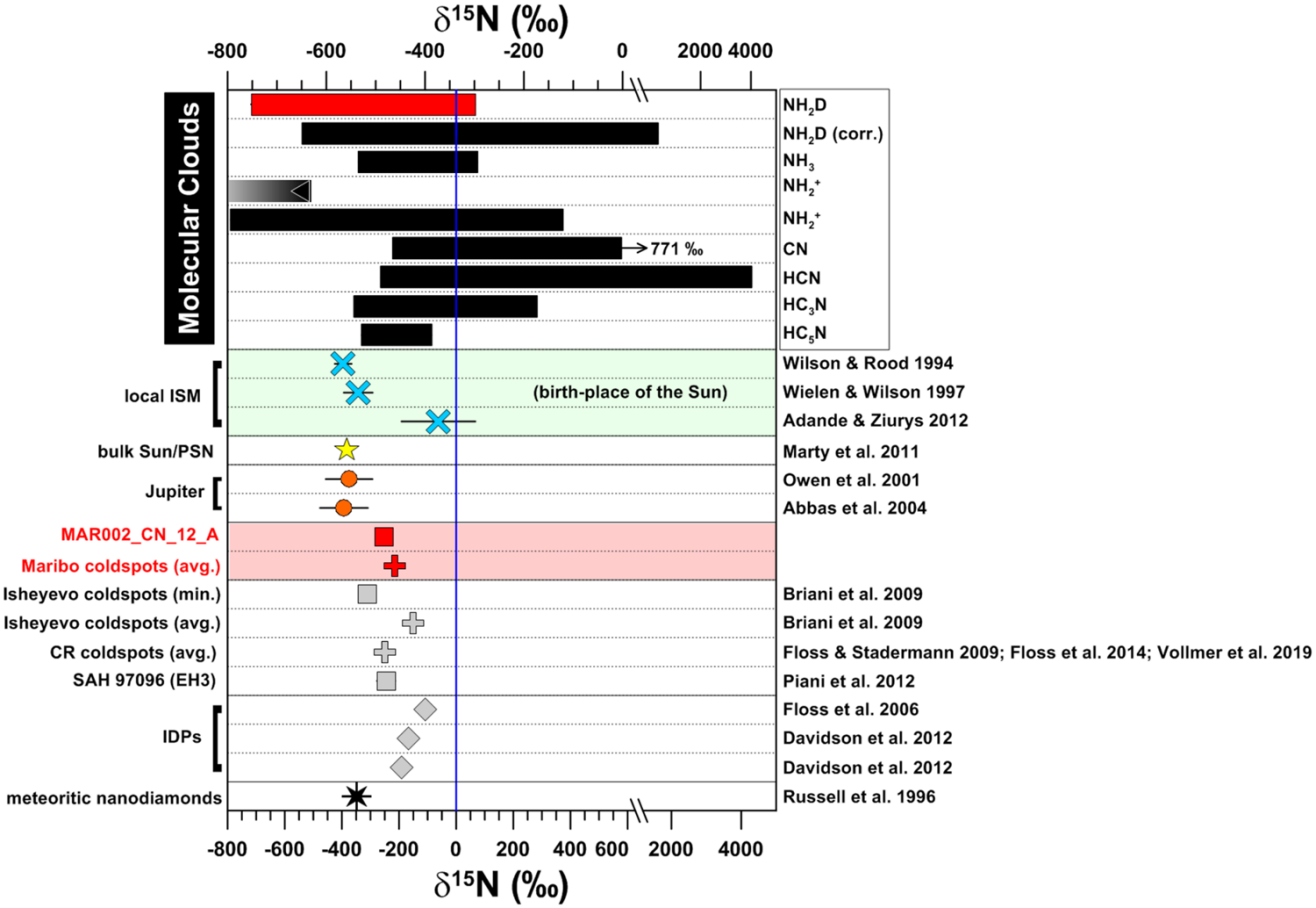
Maribo OM data with δ^15^N_air_ < 0 compared to different solar system, molecular cloud, and interstellar reservoirs. Meteorite and IDP data have been obtained by NanoSIMS, other data spectroscopically.

Figure 1 shows ion isotope ratios of 138 OM grains within Maribo obtained by NanoSIMS ion imaging compared with other primitive OM components found within carbonaceous chondrites and IDPs. Nitrogen and carbon isotopic ratios of ten ^15^N-depleted grains, together with CN^-^/C^-^ ratios are summarized in Table 1. The nitrogen isotopic compositions of these organic grains in Maribo are depleted in ^15^N compared to the terrestrial value (“coldspots”, δ^15^N_air_ ~ -200 ‰). This is a rare feature of OM in primitive meteorites as OM found in CR and CM chondrites as well as IDPs is predominantly enriched in ^15^N (Fig. 1). Moreover, their nitrogen isotopic ratios are close to values found in the local interstellar medium^19–22^ and overlap with the ranges observed for several molecular clouds^23–33^. They also point towards the solar value (δ^15^N_air_ ~ -380 ‰) obtained by analyses of solar wind samples of the Genesis mission^34^ and the composition of Jupiter´s atmosphere^35–37^ (Fig. 2). Carbon isotopic compositions of the organic grains are mostly close to the solar value within errors and are therefore not distinctive enough for further discussion here. However, we note that they also agree within errors with carbon isotopic compositions in the interstellar medium^19,20^.

**Table 1:** Carbon and nitrogen isotopic compositions of the investigated organic grains with δ^15^N_air_ values < 0, together with ^12^C^14^N^–^/^12^C^–^ ratios. Relative errors for ^12^C^14^N^–^/^12^C^–^ are < 0.009 (based on counting statistics); for the δ^15^N-values, we estimate an additional uncertainty of ~ 15 ‰ due to unknown matrix effects for the OM. Isotopic ratios are given in per mil; reported errors are 1 sigma.

Grain	δ^13^C (‰)	δ^15^N (‰)	^12^C^14^N^–^/^12^C^–^	dia. (nm)*
MAR002_CN_04_E	34 ± 20	–144 ± 29	1.37	400 ± 40
MAR002_CN_12_A	–44 ± 3	–252 ± 4	1.19	2780 ± 40
MAR002_CN_12_B	–32 ± 4	–145 ± 6	1.12	1830 ± 40
MAR002_chain_N_A3_1_B	–11 ± 24	–195 ± 29	1.89	350 ± 40
MAR002_chain_N_A3_3_C	38 ± 24	–220 ± 34	0.90	430 ± 40
MAR002_chain_N_A3_5_B	3 ± 14	–160 ± 28	0.55	510 ± 40
MAR002_chain_N_A3_6_F	47 ± 22	–138 ± 44	0.76	350 ± 40
MAR002_chain_N_A3_7_C	19 ± 20	–233 ± 26	0.94	510 ± 40
MAR002_CN_28_02_E	–18 ± 20	31	1.09	480 ± 40
MAR002_CN_29_02_F	11 ± 31	–220 ± 44	1.26	310 ± 40

*All given diameters represent *recalculated* values, which were determined from respective areas of the (typically non-circular) organic grains by assuming a circular shape for means of comparison. Given errors are based on the assumption of an uncertainty of ± 1 pixel when determining the outline of the grain and represent the pixel size for the respective secondary ion image.

**Figure 3:**
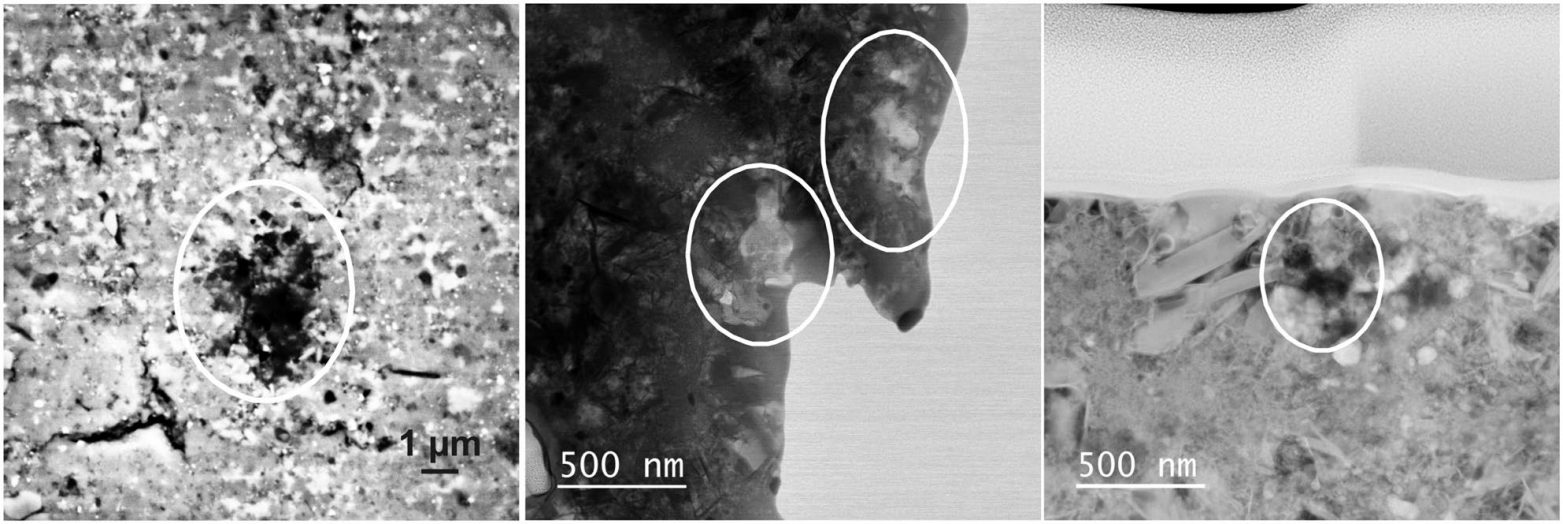
Electron microscopy images of OM morphologies within Maribo. *Left*: SEM-BSE image of a “coldspot” within the Maribo matrix. *Middle*: STEM-BF image of the small, diffuse particles (encircled) at the rim of the FIB lamella. *Right*: STEM-HAADF image of the organic particles (encircled) associated with tiny sulfides (bright spots) and phyllosilicates to the left.

SEM observations of the organic "coldspots" show that they are irregular to roundish objects that consist of smaller particles (Fig. 3). We extracted one section by the focused ion beam (FIB) technique from the area including the ^15^N-depleted OM in Fig. 3. Scanning TEM (STEM) investigations demonstrate that the OM is present as finely dispersed particles within the phyllosilicate-rich matrix (Fig. 3) with sizes generally < 500 nm. The grains exhibit a highly irregular morphology, distinct from the globular organics also found in CR and CM chondrites^2,6^. It has been demonstrated that both globular and irregular OM occur within CM and CR chondrites as well as in cometary samples, however more chemical and isotopic information exists about the globular fraction and so-called nanoglobules^8,15^. This sampling bias can be explained by the fact that the more distinct, globular or even multiglobular grains are easier to recognize within matrix regions than the diffuse OM fraction and therefore also easier to extract by FIB. However, the diffuse fraction of extraterrestrial OM represents a key component to understanding its complex evolution, particularly as it might be related to the soluble OM present within meteorites^2,4,6^.

**Figure 4:**
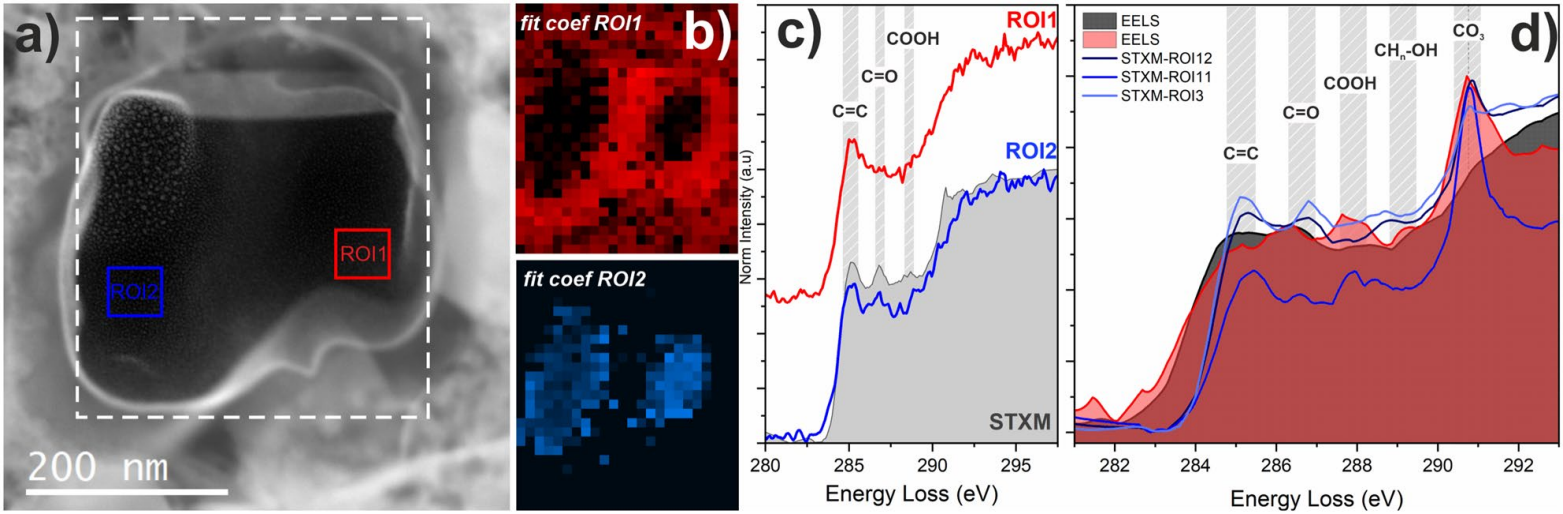
Electron energy loss spectra obtained on different parts of one organic grain. (**a**) HAADF-STEM image of the grain used for EELS measurement marked with the white rectangle, and two regions of interest marked as ROI1 and ROI 2, respectively. (**b**) Colored maps, corresponding to fit coefficients of the two spectra, showing the small-scale heterogeneity of the organic matter chemistry. (**c**) Background subtracted C-K edge EEL spectra acquired from the corresponding ROIs, plotted against a STXM spectrum for reference. Both show a prominent aromatic bonding feature at 285 eV. The spectrum corresponding to ROI2 shows an additional feature at 286.6 eV corresponding to ketone/carbonyl functional chemistry. (**d**) Comparison of EELS and STXM data demonstrating the reliable fit of the two techniques.

**Figure 5:**
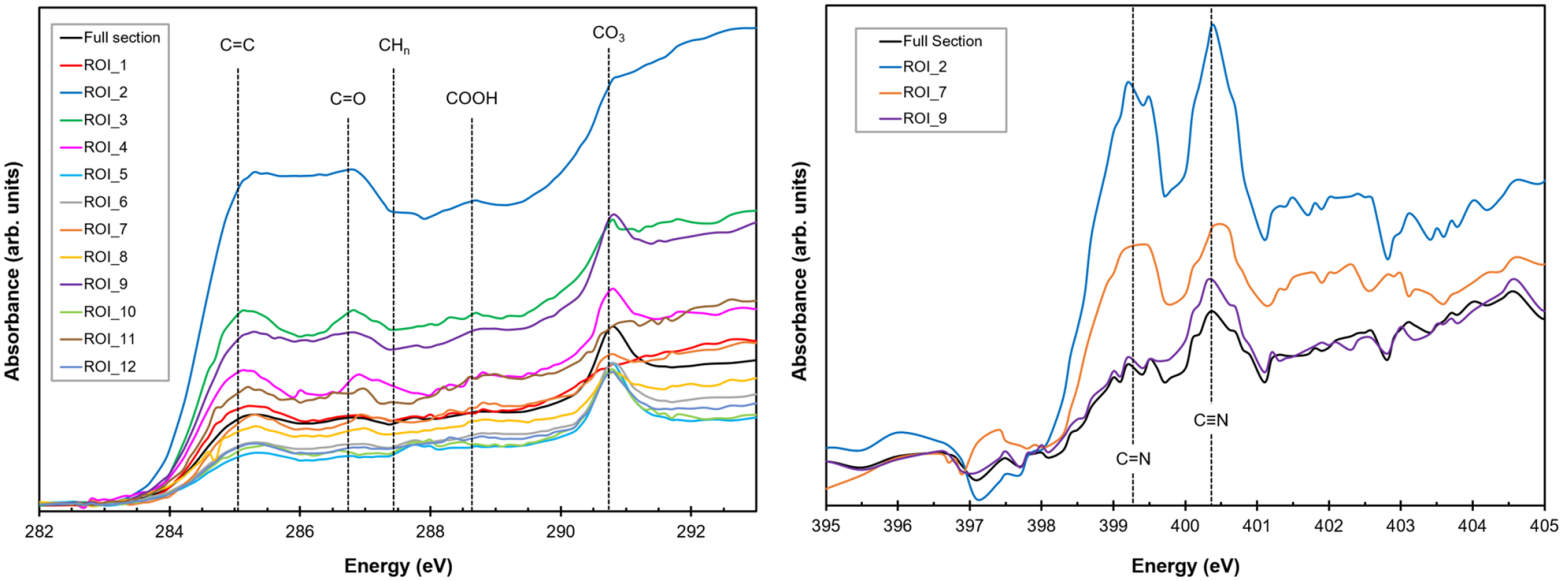
STXM spectra obtained from both all the pixels and specific regions of interest (ROI) within the FIB lamella (see also Fig. S1). *Left*: Spectra obtained at the C-K edge giving similar results as the EEL spectra, but with a high signal-to-noise ratio and more spectral detail. See text for details of detected bands. *Right*: N-K edge spectra on these organics showing the two dominant bands due to imine/nitrile C-N bonding environments.

Electron energy loss spectra obtained on different parts within the dispersed OM particles demonstrate that the functional chemistry of this material is heterogeneous within small areas of these particles down to the nanometer scale (Fig. 4a). We observe C-K edge absorption bands indicative of aromatic/olefinic carbon at around 285 eV and absorption at around 286.6 eV due to aromatic carbonyl/ketone, i.e., C-O bonding environments. Furthermore, the intensities of these bands vary among the particles on a nanometer scale, even within the same particle (Fig. 4b,c). This attests to the extremely complex chemical makeup of this OM. We confirmed the presence of N within the same particles through the N-K edge absorption features, but the low signal levels due to very low abundance within this material (N/C < 0.05) preclude any unambiguous spatial mapping of its distribution across the grains. Further analyses by scanning transmission X-ray microscopy (STXM) show excellent agreement with EELS (Fig. 4d), with more intense absorption bands, but at the cost of a loss of spatial information. STXM and EELS analyses of the Maribo OM at the C-K edge show that the 285.1 eV (aromatic) and 286.6 eV (carbonyl/ketone) bonding environments can be tracked among the different parts of the dispersed particles. In agreement with the EELS data, the relative intensity of these two bands in the STXM data varies on the nanometer scale (Fig. 5). We detected generally weak carboxyl bonding at around 288.3 eV, though the intensity does vary from region to region, but we do see some indication of aliphatic C-H_n_ chains within the organic matter at an absorption energy of ~ 287.4 eV. In most, but not all, spectra, we observe a distinct peak associated with carbonate bonding at ~ 290 eV. This association of carbonate bonding with OM has been reported in other studies as well, mostly on the diffuse fraction of OM^2,4,6^. This observation is usually explained by a later fluid overprint on respective parent bodies, but it is also possible that there is a genetic relationship between these materials and therefore an indigenous source of this carbonate bonding within the OM itself.

Further analyses at the N-K edge by STXM revealed that the functional makeup of the ^15^N-depleted nitrogen within the OM is characterized by strong C-N double and triple bonding (imine and nitrile) below the main absorption edge at around 400 eV (Fig. 5). This is clearly different to the typical functional chemistry of ^15^N-enriched OM detected within CR organics, which is more likely characterized by amine/amide (N-H_x_-) functional groups^6,18^. It should be noted in this respect that imine/nitrile functional groups are highly prone to alteration and can be easily cracked, which is why they are not very widespread among OM in different carbonaceous chondrites in general^38^.

We note that extraterrestrial OM is susceptible to modification during sample preparation (FIB) and characterization using electron/X-ray beams. In order to minimize these effects, STEM analyses were carried out using very low electron doses in the pA range. We also recorded images before, during, and after analyses, and discarded all data with observable beam damage. In addition, all spectrum images were acquired using a rastered beam with several datasets across the samples to spread the electron dose.

To summarize, our results on the primordial OM component within Maribo show that this meteorite, although clearly an aqueously altered CM chondrite breccia, contains a unique, ^15^N-depleted organic material generally not found among other carbonaceous chondrite groups. Furthermore, the functional chemistry of this material obtained both by STEM-EELS and STXM methods demonstrates that while it is similar to the most primitive OM analyzed in CR and CM chondrites that shows both strong aromatic and ketone/carbonyl features^39,40^, it differs in having less carboxylic bonding as well as indications of C-N double and triple bonding.

## Discussion

The origins of ^15^N anomalies in planetary materials are still a matter of debate^41^. The enrichment of ^15^N relative to terrestrial or solar reservoirs may be due to ion–molecule reactions at very low temperatures in the outer portions of the nascent solar nebula or interstellar space (“fractionation model”)^42,43^. Alternatively, there is growing evidence from both laboratory experiments and spectroscopic observations, that it is possible to enrich ^15^N in planetary materials by UV irradiation self-shielding in a similar manner as for oxygen (“self-shielding model”)^44–46^. According to this self-shielding model, initial/primary solar system matter was compositionally close to the Sun at δ^15^N_air_ = -383 ± 8‰^34^ and then evolved to progressively more positive values by proceeding UV interaction emanating from the young Sun. This process would result in outer solar system, i.e., cometary materials such as organics, being ^15^N-rich, dominated by HCN-NH_3/2_-ices. The complementary reservoir would then be ^15^N-poor and close to the solar and jovian compositions. The exact location of this reservoir within the solar nebula and which materials might have incorporated this component are as yet unknown.

Extraterrestrial materials with such a ^15^N-depleted signature have not been found in large quantities yet. The vast majority of OM in CI, CR, or CM chondrites and IDPs is either close to the terrestrial value or enriched in ^15^N^47^. Three ^15^N-poor “coldspots” have been found in the unequilibrated enstatite chondrite Sahara 97096^48^, as well as one peculiar ^15^N- and ^13^C-depleted organic grain (δ^13^C = -300‰, δ^15^N = -264‰) within the primitive CO 3.0 chondrite DOM 08006^49^. The isotopic composition of this grain is similar to some rare presolar graphite grains from low-metallicity asymptotic giant branch stars, but based on its N content of about 10 at. % such an origin was excluded. We can also rule out the possibility that we have detected presolar graphite grains, because the morphologies and functional chemistry of the OM grains detected here are clearly different. Within a lithic clast of the CB/CH chondrite Isheyevo, Briani et al.^50^ reported areas with similar ^15^N-depleted compositions by NanoSIMS imaging, but did not describe any further chemical information on that type of material. Furthermore, some organic grains within CR chondrites with such compositions have been identified (Fig. 1), but no chemical or morphological information was presented. Recently, solar silicon nitride grains from different meteorites have been measured by NanoSIMS^51^, which are also depleted in ^15^N relative to the terrestrial value, but nitrogen in those grains is still heavier than that of the Sun or the Maribo organics detected here. Finally, bulk isotopic analyses of nanodiamond separates from carbonaceous, ordinary, and enstatite chondrites have resulted in ^15^N-depleted isotopic compositions^52^, similar to that of the solar wind. However, the origins of meteoritic nanodiamonds are still a matter of debate, because both a solar and presolar heritage is possible^53^. Nevertheless, the carbonaceous precursor material of some of these nanodiamonds, which might have condensed by a chemical vapor deposition-like process^54^ close to the Sun, is possibly related to this ^15^N-depleted reservoir.

All of these rare observations of ^15^N-depleted materials indicate that the different nitrogen reservoirs within the solar system are relatively well mixed, because the majority of meteorites and the Earth plot in between the solar and cometary endmembers. It was also proposed that this mixing process was rather quick, because the self-shielding model predicts that the ^15^N enrichment occurred within the first 1000 years of solar system formation^55^. However, an apparently contradictory idea states that there is a gradient in N/C as well as in nitrogen isotopic composition within the early solar nebula^41,56^. Whereas materials with N-poor and ^15^N-depleted compositions accreted close to the Sun, N-rich and ^15^N-enriched materials are found in the outer solar system. Recently, isotopic analyses of nitrides from different chondrites generally supported such a sequence^57^, and a similar gradient has been observed around other stellar systems^22^. It is also possible that these materials can be distributed to different locations around the solar system, based on the observation of a ^15^N-rich CAI in a meteorite^58^. It is also interesting to note in this respect that ^15^N-depleted OM has not been reported yet within primitive IDPs, which are thought to originate in cometary, i.e., outer solar system bodies. However, some bulk ^15^N-depleted IDPs do indeed exist (Fig. 1). Spectroscopically, no comets have been detected with this ^15^N-depleted signature as well.

Whereas some chemical data exists about the ^15^N-enriched reservoir from both bulk and in-situ analyses of meteoritic and cometary OM, there is almost no information available about dust species or the functional chemistry of the ^15^N-depleted nitrogen reservoir. It has been suggested that this ^15^N-depleted reservoir is preferentially retained by reduced phases such as metal or silicates^59^ or refractory phases such as nitrides^35,51^. It is also possible, based on observations in the ISM, that ^15^N-poor ammonia is an important reactive agent^24^, which would likely result in ^15^N-depleted organics in the ISM or the solar nebula. However, recent hypotheses also identify ^15^N-enriched ammonia within ices as an important constituent to modify asteroidal organics^6,60^. Therefore, nitrogen isotopic compositions of ammonia span a wide range from very negative to very positive δ^15^N values including reactive ammonia with terrestrial isotopic composition^61^. Thus, this molecule might have played an important role for the formation of organics presented here. But even though the candidates containing this primordial nitrogen isotopic signature are controversially debated, detailed analyses of such grains are still lacking, specifically about the functional chemistry of such phases. These investigations are crucial to understand the evolution of nitrogen isotopes within the solar system, but also around other stellar systems.

Here we show that the ^15^N-depleted material detected within Maribo is characterized by OM with a carbon functional chemistry that is assumed to represent more primordial organics found, for example, within CR chondrites. Such OM is generally characterized by three major functional groups in absorption spectra, i.e., aromatic, carbonyl/ketone, and carboxylic bonding environments, also referred to as “IOM-like” as well as some aliphatic carbon chains. This distinct functional chemistry might then change its relative abundance in response to proceeding alteration reactions in different parent body environments.

If we compare this unique material to diffuse OM analyzed by high-spatial resolution STXM methods within primitive lithologies of the Paris and Murchison CM chondrites^39^, it can be shown that those grains generally contain more abundant carboxylic functional groups and fewer ketone/carbonyl units (see Fig. 8 in Vinogradoff et al.^39^). Organic matter in less altered areas of those CM chondrites, on the contrary, shows less abundant carboxylic functional groups, so similar to the signatures reported here. Although there are indications that carboxylic functional groups might also be present in the primordial OM of CR chondrites, there is growing evidence that the abundance of these carboxylic bonding groups increases with proceeding fluid reactions on the parent body^6,40^. Furthermore, the detection of more abundant aliphatic chains in comparison to aromatic units in extracted IOM might as well attest to less alteration, because Paris, a generally less altered carbonaceous chondrite, has a higher aliphatic/aromatic ratio than IOM in Murchison^39^. This is in agreement with the generally less altered character of our OM that also shows some indications of aliphatic chains in EELS and STXM spectra. The observed C-K edge functionality of this diffuse Maribo OM is therefore more similar to the more primitive OM in generally less altered CR and CM chondrite lithologies showing aromatic and ketone/carbonyl functional groups, some aliphatic chains, and little carboxylic bonding^40^.

Furthermore, in the majority of OM analyzed in the most primitive CR chondrite lithologies, no N-K edge functionality can be detected at all, because it appears that this component is quite reactive to aqueous alteration and can be easily redistributed. In those rare cases, where the N-K edge functionality could be analyzed, it was shown that it contains more amine/amide-like moieties (N-H_x_) indicating fluid reactions with reactive ammonia, while still maintaining its anomalous isotopic composition^6,18^. However, C-N double and triple bonding (imine and nitrile) is generally rarely detected, because they break up rapidly upon aqueous reactions and are easily hydrogenated. This has been demonstrated by Cody and Alexander^38^, who show that these functional chemistry fingerprints are almost absent within the OM in the more heavily altered lithologies of Tagish Lake (C2_ung_). Thus, in the case of the ^15^N-depleted diffuse Maribo OM, the combination of highly aromatic carbon bonding environments, very few carboxylic functional groups, and the detection of imine/nitrile functional groups that are usually prone to alteration support the primordial chemical character of this OM. We conclude that the Maribo OM investigated here has sampled a ^15^N-depleted carbonaceous reservoir that is dominated by carbon and nitrogen functional chemistry signatures that have been recently assigned to more primordial organics in primitive CR and CM lithologies. The origin of this reservoir is difficult to assess from our data, because the CM parent body, from which Maribo stems, likely accreted material in the outer solar system, maybe at about 3.8 A.U. beyond the initial orbit of Jupiter^62^. However, the different components within this highly complex breccia can have diverse origins within the solar nebula, because it is well known, by analyses of Wild 2 samples for example, that different materials were distributed across the disk at the early stages of solar nebula evolution^62^. It is probable that the ^15^N-poor material did not originate in the inner solar nebula, although the proposed ^15^N gradient would indicate this origin, because fragile organics would not survive intense UV irradiation from the young Sun. This might be the reason why such a ^15^N-depleted signature is usually detected within more refractory phases such as nitrides, which are more resistant to high temperatures (> > 1000 K) and extreme irradiation conditions. However, it is interesting to note that the δ^15^N value of -200 ‰ in Maribo OM is close to that of HCN molecules detected within the ISM^19–21,41^. We therefore speculate here that the OM detected in this work represents material originating in the interstellar medium, where abundant organic molecules such as polycyclic aromatic hydrocarbons have been detected. Interestingly, the functional chemistry is similar to organics detected within ultra-carbonaceous Antarctic micrometeorites thought to originate in the outer solar nebula^56^, although those organics are usually ^15^N-rich. This can be explained by the fact that both isotopic signatures of OM are present within solar system samples, but that the ^15^N-depleted material is much less abundant. We therefore conclude that Maribo represents one of the key CM chondrite breccias present in our collection and has sampled primordial OM probably originating in the interstellar medium.

## Methods

### Nano-secondary ion mass spectrometry (NanoSIMS)

Matrix areas of a polished and carbon-coated Maribo thin section (PL09090) were analyzed with the NanoSIMS 50 at the MPIC in Mainz, where a ~ 100 nm Cs^+^ primary ion beam (~ 1 pA) was rastered over selected sample areas (8 × 8 µm^2^ in size). We acquired secondary ion images of ^12,13^C^–^, ^12^C^14^N^–^, ^12^C^15^N^–^, and ^28^Si^–^ in multi-collection mode. Mass resolution was sufficient (m/Δm > 8000, CAMECA-definition, using a 10%-90% slope at mass 26) to separate ^13^C^–^ from ^12^C^1^H^–^, and ^12^C^15^N^–^ from ^13^C^14^N^–^. As ^11^B^16^O^–^ is not fully separated from the ^12^C^15^N^-^ peak, measurements were performed -0.8 V to the left of the ^12^C^15^N^-^ peak center. Prior to analysis, all areas were sputtered with a high current primary beam (~ 20 pA) to remove the carbon coating and implant primary ions for efficient secondary ion extraction. Carbon and nitrogen isotopic ratios were normalized to a synthetic N-doped SiC standard of known C-isotopic composition (δ^13^C_PDB_ = -29 ‰; ^13^C/^12^C_PDB_ = 0.011237) and assumed N-isotopic composition of terrestrial air (δ^15^N_air_ = 0; ^15^N/^14^N_air_ = 3.676 × 10^−3^). We estimate an uncertainty of ~ 15 ‰ for our N-isotope measurements of the OM (of unknown composition) due to matrix effects adding to the pure Poisson error from counting statistics (see also Leitner et al. 2018^51^ for further details). For data reduction and processing, we used in-house software developed at the MPIC. Deviations of the isotopic compositions from the respective standard ratios are reported as δ-values displaying the deviation in per mil from the standard.

### Scanning electron microscopy (SEM)

Identified “coldspots” were documented by SEM on a LEO 1530 field emission gun scanning electron microscope at MPIC. The main detection signal was backscattered electron (Z) contrast using a high-resolution in-lens detector, where OM can be clearly identified by its darker contrast compared to surrounding matrix.

### Aberration-corrected scanning TEM (UltraSTEM)

An electron-transparent lamella of one ^15^N-depleted organic grain was prepared with the FIB technique using a FEI/ThermoScientific Nova 600 Nanolab Dual-Beam Workstation at the MPI for Polymer Research (MPIP) in Mainz. For STEM-EELS analyses at the carbon and nitrogen edges, we used a dedicated aberration-corrected Nion UltraSTEM100MC–Hermes operated at 60 kV in so-called “gentle” STEM conditions (low acceleration voltage to avoid knock-on damage to carbon-based material, ultra-high vacuum conditions to prevent chemical etching of the sample). The instrument is equipped with a cold field emission electron source with a nominal energy spread of around 0.3 eV (as measured by the FWHM of the ZLP). The microscope features an ultra-stable stage, conventional BF and HAADF detectors and a Gatan Enfinium ERS energy loss spectrometer. The probe forming optics were adjusted to provide a 0.09 nm probe with a beam convergence of 30 mrad (half-angle), while a collection half-angle of 42 mrad was chosen for EELS analysis. EEL spectra were acquired in Dual EELS mode, which allows for quasi-simultaneous acquisition of low- and core-loss energy spectra; other EELS acquisition parameters were further tailored to obtain electron doses low enough to avoid any observable beam damage. For more details regarding analysis see Vollmer et al. (2020)^6^. Energy spectra from regions of interest were energy-calibrated with respect to the exact position of the ZLP and the energy offset compared to spectra from a graphite standard sample, acquired under identical conditions. The spectra were background subtracted using a decaying power-law function. The colored maps in Fig. 4 were produced using multiple linear least squares (MLLS) fitting of extracted spectra.

### Scanning transmission X-ray microscopy (STXM)

Scanning transmission X-ray microscopy (STXM) and X-ray absorption near-edge structure (XANES) measurements were performed at the I08 beamline at Diamond Light Source (DLS), UK. Spatially correlated energy dependent image stacks (~ 5 × 3 µm, 100 × 60 pixels) were acquired at the carbon (278–310 eV) and nitrogen (390–420 eV) K edges with a nominal beam size of ~ 70 nm and a dwell time per pixel of 10 ms. For both the carbon and nitrogen measurements, the step size was 0.1 eV over the main edge and where important diagnostic features occur, and between 0.2–0.5 eV across the rest of the scan. The MANTiS program (Lerotic et al. 2014) was used to align the image stacks, normalize to the background X-ray intensity (I_0_), and finally extract XANES spectra from every pixel in the full FIB lamella and specific regions of interest (ROIs).

## Supplementary information

Supplementary information.

## Data Availability

Correspondence and requests for materials should be addressed to C.V.

## References

[1] Alexander CMOD, Cody GD, De Gregorio BT, Nittler LR, Stroud RM (2017). The nature, origin and modification of insoluble organic matter in chondrites, the major source of Earth’s C and N. Chem. Erde.

[2] Le Guillou C, Bernard S, Brearley AJ, Remusat L (2014). Evolution of organic matter in Orgueil, Murchison and Renazzo during parent body aqueous alteration: In situ investigations. Geochim. Cosmochim. Acta.

[3] Hopp T, Vollmer C (2017). Chemical composition and iron oxidation state of amorphous matrix silicates in the carbonaceous chondrite Acfer 094. Meteorit. Planet. Sci..

[4] Vollmer C (2014). Fluid-induced organic synthesis in the solar nebula recorded in extraterrestrial dust from meteorites. Proc. Natl. Acad. Sci..

[5] Floss C, Stadermann FJ (2009). High abundances of circumstellar and interstellar C-anomalous phases in the primitive CR3 chondrites QUE 99177 and MET 00426. Astrophys J.

[6] Vollmer C (2020). Isotopic compositions, nitrogen functional chemistry, and low-loss electron spectroscopy of complex organic aggregates at the nanometer scale in the carbonaceous chondrite Renazzo. Meteorit. Planet. Sci..

[7] Floss C, Le Guillou C, Brearley AJ (2014). Coordinated NanoSIMS and FIB-TEM analyses of organic matter and associated matrix materials in CR3 chondrites. Geochim. Cosmochim. Acta.

[8] De Gregorio BT (2010). Isotopic anomalies in organic nanoglobules from Comet 81P/Wild 2: comparison to Murchison nanoglobules and isotopic anomalies induced in terrestrial organics by electron irradiation. Geochim. Cosmochim. Acta.

[9] Floss C (2006). Identification of isotopically primitive interplanetary dust particles: a NanoSIMS isotopic imaging study. Geochim. Cosmochim. Acta.

[10] Davidson J, Busemann H, Franchi IA (2012). A NanoSIMS and Raman spectroscopic comparison of interplanetary dust particles from comet Grigg-Skjellerup and non-Grigg Skjellerup collections. Meteorit. Planet. Sci..

[11] Hewins RH (2014). The Paris meteorite, the least altered CM chondrite so far. Geochim. Cosmochim. Acta.

[12] Leroux H, Cuvillier P, Zanda B, Hewins RH (2015). GEMS-like material in the matrix of the Paris meteorite and the early stages of alteration of CM chondrites. Geochim. Cosmochim. Acta.

[13] Bischoff A (1998). Aqueous alteration of carbonaceous chondrites: evidence for preaccretionary alteration—a review. Meteorit. Planet. Sci..

[14] Thomen, A. *et al.* Spatial relations between D/H and N isotopic anomalies in Orgueil and Murchison insoluble organic matter: a NanoSIMS study. *Ann. Meet. Meteorit. Soc.* **72**, abstr. #5284 (2009).

[15] De Gregorio BT (2013). Isotopic and chemical variation of organic nanoglobules in primitive meteorites. Meteorit. Planet. Sci..

[16] Haack H (2012). Maribo—a new CM fall from Denmark. Meteorit. Planet. Sci..

[17] Vollmer, C. *et al.* The early stages of aqueous alteration in CM chondrites—TEM-UltraSTEM-STXM investigations of the less-altered chondrite Maribo. *Lunar Planet. Sci. Conf.* **45**, #1354 (2014).

[18] van Kooten EMME (2018). Isotope record of mineralogical changes in a spectrum of aqueously altered CM chondrites. Geochim. Cosmochim. Acta.

[19] Wilson TL, Rood RT (1994). Abundances in the interstellar medium. Ann. Rev. Astron. Astrophys..

[20] Wielen R, Wilson TL (1997). The evolution of the C, N, and O isotope ratios from an improved comparison of the interstellar medium with the Sun. Astron. Astrophys..

[21] Adande GR, Ziurys LM (2012). Millimeter-wave observations of CN and HNC and their ^15^N isotopologues: a new evaluation of the ^14^N/^15^N ratio across the Galaxy. Astrophys. J..

[22] Hily-Blant P, Magalhaes de Souza V, Kastner J, Forveille T (2019). Multiple nitrogen reservoirs in a protoplanetary disk at the epoch of comet and giant planet formation. Astron. Astrophys..

[23] Gerin M (2009). Detection of ^15^NH^2^D in dense cores: a new tool for measuring the ^14^N/^15^N ratio in the cold ISM. Astron. Astrophys..

[24] Lis DC, Wootten A, Gerin M, Roueff E (2010). Nitrogen isotopic fractionation in interstellar ammonia. Astrophys. J..

[25] Daniel F (2013). Nitrogen isotopic ratios in Barnard 1: a consistent study of the N_2_H+, NH_3_, CN, HCN, and HNC isotopologues. Astron. Astrophys..

[26] Daniel F (2016). N_2_H^+^ and N^15^NH^+^ toward the prestellar core 16293E in L1689N. Astron. Astrophys..

[27] Redaelli E (2018). ^14^N/^15^N ratio measurements in prestellar cores with N_2_H^+^: new evidence of ^15^N-antifractionation. Astron. Astrophys..

[28] Kahane C (2018). First measurement of the ^14^N/^15^N ratio in the analog of the sun progenitor OMC-2 FIR4. Astrophys. J..

[29] Magalhaes VS, Hily-Blant P, Faure A, Hernandez-Vera M, Lique F (2018). Abundance of HCN and its C and N isotopologues in L1498. Astron. Astrophys..

[30] Liszt HS, Ziurys LM (2012). Carbon isotope fractionation and depletion in TMC1. Astrophys. J..

[31] Araki M (2016). Precise observations of the ^12^C/^13^C ratios of HC_3_N in the low-mass star-forming region L1527. Astrophys. J..

[32] Taniguchi K, Saito M (2017). First detection of HC_5_^15^N in the interstellar medium. Pub. Astron. Soc. Jpn..

[33] Hily-Blant P (2018). The nitrogen isotopic ratio of HC_3_N towards the L1544 prestellar core. Mon. Not. R. Astron. Soc..

[34] Marty B, Chaussidon M, Wiens RC, Jurewicz AJG, Burnett DS (2011). A 15N-poor isotopic composition for the solar system as shown by genesis solar wind samples. Science.

[35] Meibom A (2007). Nitrogen and carbon isotopic composition of the sun inferred from a high-temperature solar nebula condensate. Astrophys. J..

[36] Owen T, Mahaffy PR, Niemann HB, Atreya S, Wong M (2001). Protosolar nitrogen. Astrophys. J..

[37] Abbas MM (2004). The nitrogen isotopic ratio in Jupiter’s atmosphere from observations by the composite infrared spectrometer on the Cassini spacecraft. Astrophys. J..

[38] Cody, G. D. & Alexander, C. M. O`D. The peculiar nature of nitrogen in organic solids from chondritic meteorites. *Lunar Planet. Sci. Conf.* **48**, #2747 (2017).

[39] Vinogradoff V (2017). Paris vs. Murchison: impact of hydrothermal alteration on organic matter in CM chondrites. Geochim. Cosmochim. Acta.

[40] Changela HG, Le Guillou C, Bernard S, Brearley AJ (2018). Hydrothermal evolution of the morphology, molecular composition, and distribution of organic matter in CR (Renazzo-type) chondrites. Meteorit. Planet. Sci..

[41] Füri E, Marty B (2015). Nitrogen isotope variations in the solar system. Nat. Geosci..

[42] Terzieva R, Herbst E (2000). The possibility of nitrogen isotopic fractionation in interstellar clouds. Mon. Not. R. Astron. Soc..

[43] Wirström ES, Charnley SB, Cordiner MA, Milam SN (2012). Isotopic anomalies in primitive solar system matter: spin-state-dependent fractionation of nitrogen and deuterium in interstellar clouds. Astrophys J.

[44] Chakraborty S (2014). Massive isotopic effect in vacuum UV photodissociation of N_2_ and implications for meteorite data. Proc. Natl. Acad. Sci..

[45] Muskatel BH, Remacle F, Thiemens MH, Levine RD (2011). On the strong and selective isotope effect in the UV excitation of N_2_ with implications toward the nebula and Martian atmosphere. Proc. Natl. Acad. Sci..

[46] Visser R (2018). Nitrogen isotope fractionation in protoplanetary disks. Astron. Astrophys..

[47] Alexander CMOD, Fogel M, Yabuta H, Cody GD (2007). The origin and evolution of chondrites recorded in the elemental and isotopic compositions of their macromolecular organic matter. Geochim. Cosmochim. Acta.

[48] Piani L (2012). Structure, composition, and location of organic matter in the enstatite chondrite Sahara 97096 (EH3). Meteorit. Planet. Sci..

[49] Nittler LR (2018). High abundances of presolar grains and 15N-rich organic matter in CO3.0 chondrite Dominion Range 08006. Geochim. Cosmochim. Acta.

[50] Briani G (2009). Pristine extraterrestrial material with unprecedented nitrogen isotopic variation. Proc. Natl. Acad. Sci. USA.

[51] Leitner J, Vollmer C, Henkel T, Ott U, Hoppe P (2018). An isotopic, elemental and structural study of silicon nitride from enstatite chondrites. Geochim. Cosmochim. Acta.

[52] Russell SS, Arden JW, Pillinger CT (1996). A carbon and nitrogen isotope study of diamond from primitive chondrites. Meteorit. Planet. Sci..

[53] Dai ZR (2002). Possible in situ formation of meteoritic nanodiamonds in the early solar system. Nature.

[54] Daulton TL, Eisenhour DD, Bernatowicz TJ, Lewis RS, Buseck PR (1996). Genesis of presolar diamonds: comparative high-resolution transmission electron microscopy study of meteoritic and terrestrial nano-diamonds. Geochim. Cosmochim. Acta.

[55] Lyons JR (2009). N_2_ self-shielding in the solar nebula. Meteorit. Planet. Sci..

[56] Dartois E (2018). Dome C ultracarbonaceous antarctic micrometeorites—infrared and raman fingerprints. Astron. Astrophys..

[57] Leitner, J. *et al.* Investigation of nitrides in carbonaceous chondrites: a window to early solar nebula processes? *Lunar Planet. Sci. Conf.* **51**, Abstr. #1937 (2020).

[58] Füri E, Chaussidon M, Marty B (2015). Evidence for an early nitrogen isotopic evolution in the solar nebula from volatile analyses of a CAI from the CV3 chondrite NWA 8616. Geochim. Cosmochim. Acta.

[59] Marty B (2010). Nitrogen isotopes in the recent solar wind from the analysis of genesis targets: evidence for large scale isotope heterogeneity in the early solar system. Geochim. Cosmochim. Acta.

[60] Pizzarello S, Bose M (2015). The path of reduced nitrogen toward early Earth: the cosmic trail and its solar shortcuts. Astrophys. J..

[61] Harries D, Hoppe P, Langenhorst F (2015). Reactive ammonia in the solar protoplanetary disk and the origin of Earth’s nitrogen. Nat. Geosci..

[62] Desch SJ, Kalyaan A, Alexander CMOD (2018). The effect of Jupiter's formation on the distribution of refractory elements and inclusions in meteorites. Astrophys. J. Suppl. Ser..

