# Supplementary material for: A primordial 15N-depleted organic component detected within the carbonaceous chondrite Maribo
Source: Sci Rep. 2020 Nov 20;10:20251. doi: 10.1038/s41598-020-77190-z (PMC7679378; doi:10.1038/s41598-020-77190-z)
Supplement: Supplementary file 1 — Supplementary information. [file 41598_2020_77190_MOESM1_ESM.doc]

***Supplementary Information for***

**A primordial ^15^N-depleted organic component detected within the carbonaceous chondrite Maribo**

*Christian Vollmer^1*^, Jan Leitner^2^, Demie Kepaptsoglou^3,4^, Quentin M. Ramasse^3,5^, Ashley J. King^6,7^, Paul F. Schofield^7^, Addi Bischoff^8^, Tohru Araki^9^, and Peter Hoppe^2^*

^1^Institut für Mineralogie, Westfälische Wilhelms-Universität, Corrensstr. 24, 48149 Münster, Germany

^2^Max Planck Institute for Chemistry, Particle Chemistry Department, Hahn-Meitner-Weg 1, 55128 Mainz, Germany

^3^SuperSTEM Laboratory, Keckwick Lane, Daresbury, UK

^4^Jeol Nanocentre and Department of Physics, University of York, Heslington, YO10 5DD, UK

^5^School of Chemical and Process Engineering and School of Physics and Astronomy, University of Leeds, Leeds LS2 9JT, UK

^6^School of Physical Sciences, The Open University, Milton Keynes, MK7 6AA, UK

^7^Planetary Materials Group, Department of Earth Sciences, Natural History Museum, London, SW7 5BD, UK

^8^Institut für Planetologie, Westfälische Wilhelms-Universität, Wilhelm-Klemm-Str. 10, 48149 Münster, Germany

^9^Diamond Light Source, Didcot, OX11 0DE, UK.

*Corresponding author: Phone: +492518333461 Email: christian.vollmer@wwu.de


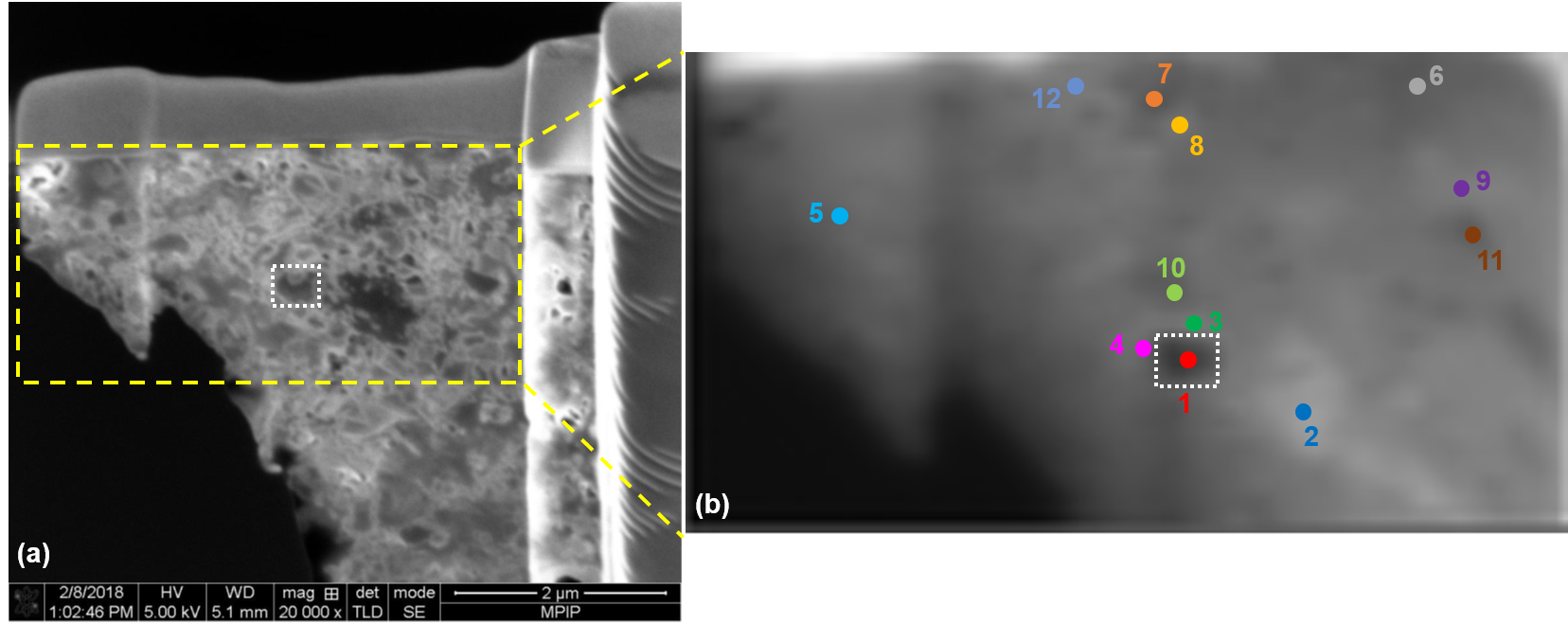


**Fig. S1.** Electron (a) and corresponding STXM image at 285 eV (b) of a Maribo FIB section showing the ROIs from where spectra presented in Fig. 5 were extracted. The dashed white box indicates the location of the organic grain shown in Fig. 4.
